# Repotrectinib: Redefining the therapeutic landscape for patients with *ROS1* fusion‐driven non‐small cell lung cancer

**DOI:** 10.1002/ctm2.70017

**Published:** 2024-10-14

**Authors:** Antoine Desilets, Matteo Repetto, Alexander Drilon

**Affiliations:** ^1^ Department of Medicine Early Drug Development Service Memorial Sloan Kettering Cancer Center New York New York USA; ^2^ Department of Oncology and Hemato‐Oncology University of Milan Milan Italy; ^3^ Department of Medicine Weill Cornell Medicine and New York Presbyterian Hospital New York New York USA

**Keywords:** non‐small cell lung cancer, repotrectinib, ROS1 fusions, tyrosine kinase inhibitors

## Abstract

The ROS1 proto‐oncogene encodes a receptor tyrosine kinase with structural homology to other oncogenic drivers, including ALK and TRKA‐B‐C. The FDA‐approved tyrosine kinase inhibitors (TKIs) crizotinib and entrectinib have demonstrated efficacy in treating ROS1 fusion‐positive NSCLC. However, limitations such as poor blood‐brain barrier penetration and acquired resistance, particularly the ROS1 G2032R solvent‐front mutation, hinder treatment durability. Repotrectinib, a next‐generation macrocyclic TKI, was rationally designed to overcome on‐target resistance mutations and improve brain distribution through its low molecular weight. In the TRIDENT‐1 clinical trial, repotrectinib demonstrated significant efficacy in both TKI‐naïve and TKI‐pretreated patients with ROS1‐rearranged NSCLC, including those with CNS metastases and G2032R resistance mutations. In the TKI‐naïve cohort (*n* = 71), 79% of patients achieved an objective response, with a median progression‐free survival (PFS) of 35.7 months, surpassing all previously approved ROS1 TKIs. In patients who had received one prior ROS1 TKI but were chemotherapy‐naïve (*n* = 56), objective responses were observed in 38%, and median PFS was 9.0 months. The safety profile of repotrectinib was consistent with earlier‐generation ROS1 TKIs and common adverse events included anemia, neurotoxicity, increased creatine kinase levels, and weight gain. These findings underscore the potential of repotrectinib to address unmet needs in ROS1‐rearranged NSCLC, offering durable responses and improved intracranial activity. Future research should prioritize developing next‐generation, selective ROS1 inhibitors to reduce Trk‐mediated toxicities and improve treatment tolerance.

The *ROS1* proto‐oncogene encodes an evolutionarily conserved receptor tyrosine kinase (RTK) with significant structural homology with the tyrosine kinase domains of additional oncogenic drivers, including ALK and TRKA‐B‐C.^1^, ^2^ *ROS1* rearrangements have been implicated in the tumorigenesis of multiple cancer types, including non‐small cell lung cancer (NSCLC),^3^ glioblastomas,^4^ inflammatory myofibroblastic tumors^5^ and spitzoid neoplasms.^6^ Interchromosomal translocations account for the majority of *ROS1* rearrangements in NSCLC, with *CD74* representing the dominant 5′ gene partner (involved in 44% of cases), followed by *EZR* (16%), *SDC4* (14%) and *SLC34A2* (10%).^2^ Functional ROS1 fusion proteins canonically involve an intact intracellular ROS1 kinase domain coupled to the N‐terminal domain of the associated partner gene. Previously approved tyrosine kinase inhibitors (TKIs) for the treatment of advanced *ROS1* fusion‐driven NSCLC include crizotinib^7^, ^8^ and entrectinib,^9^, ^10^ which have demonstrated meaningful benefits both in terms of response, progression‐free survival (PFS) and overall survival (OS) compared to the historical activity of chemotherapy inclusive regimens.

Several unmet needs persist in patients treated with early‐generation ROS1‐directed TKIs; these older drugs can have variable blood‐brain barrier penetration and limited durability of responses due to the development of acquired resistance (Figure 1). Central nervous system (CNS) metastases are prevalent in patients with newly diagnosed *ROS1* fusion‐positive NSCLC and represent a common site of progression on first‐line crizotinib, highlighting the importance of developing ROS1 TKIs with improved brain penetration.^11^, ^12^ Whereas entrectinib exhibits some CNS activity, intracranial responses remain modest with only a minority (11%) of patients achieving objective responses following CNS‐only progression events on crizotinib.^10^ In addition, treatment of *ROS1*‐rearranged NSCLC with first‐line crizotinib or entrectinib has been associated with on‐target resistance events, including the recurrent G2032R mutation occurring at the solvent‐front (SF) region of the ATP‐binding site.^13^, ^14^, ^15^ *ROS1* G2032R is homologous to the G1202R *ALK* kinase domain mutation which has been identified in crizotinib‐resistant *ALK*‐rearranged NSCLC.^16^, ^17^ While lorlatinib has demonstrated significant activity against *ALK* G1202R,^18^ preclinical models have predicted it lacked optimal efficacy against *ROS1* G2032R.^15^ Hence, next‐generation ROS1 inhibitors are urgently needed in clinical practice to improve patient outcomes in both the treatment‐naïve and acquired resistance settings.

**FIGURE 1:**
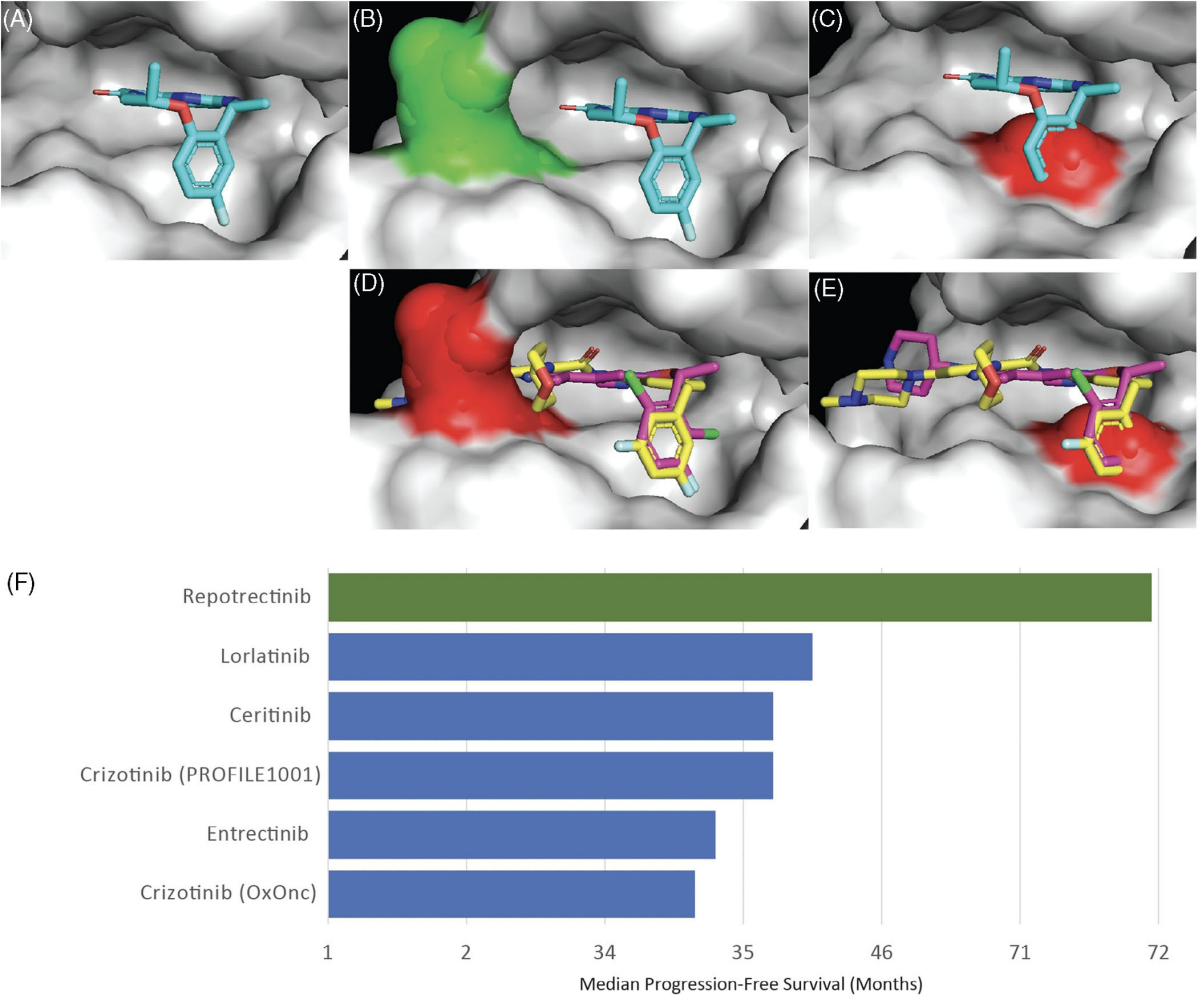
Panel A: Model of Repotrectinib (Cyan) binding mode on ROS1 (white). Panel B: Model of Repotrectinib (Cyan) successful binding mode on ROS1 G2032R (Green). Panel C: Structural basis of Repotrectinib (Cyan) steric clash with ROS1 L2086F (Red). Panel D: Model of Crizotinib (Purple) and Entrectinib (Yellow) steric clash with ROS1 G2032R (Red). Panel E: Structural basis of Crizotinib (Purple) and Entrectinib (Yellow) steric clash with cross‐resistant ROS1 L2086F (Red) mutant. Panel F: Comparison of PFS of Repotrectinib in TKI‐naïve patients with reported PFS of FDA‐approved or other guideline‐listed ROS1 TKIs.^8^, ^10^, ^23^, ^30^, ^31^, ^32^

Repotrectinib (TPX‐0005) is a highly potent, next‐generation, three‐dimensional macrocyclic TKI with activity against ROS1, TRKA‐B‐C and ALK. Repotrectinib was rationally designed to circumvent steric interference mediated by *ROS1* SF mutations, such as G2032R, with structural models predicting that drug binding was unaffected by the bulky, positive side chain of arginine (R) at the SF interface.^19^, ^20^ In *ROS1* fusion‐containing cell lines, repotrectinib more potently inhibited WT ROS1 than either entrectinib or crizotinib while maintaining meaningful activity against G2032R as well as additional on‐target mutations.^21^ Furthermore, repotrectinib has a lower molecular weight when compared to additional ROS1/ALK/TRK TKIs which is predicted to improve brain distribution.^19^ Preclinical studies first reported the high potency of repotrectinib against ROS1 and TRK, with a 15‐fold kinase specificity index when compared to ALK, as well as tumour regressions in xenograft models of both CD74‐ROS1 fusion WT and *ROS1* G2032R‐mutant tumours.^19^, ^20^, ^22^ Clinical proof‐of‐concept cases of the activity of repotrectinib were previously reported, with objective intracranial and systemic radiological responses noted in the post‐crizotinib setting in a patient with CD74‐ROS1 fusion‐positive NSCLC also harbouring a G2032R SF mutation.^19^

TRIDENT‐1 was a first‐in‐human global, multicenter, single‐arm phase 1/2 trial evaluating the safety and efficacy of repotrectinib across multiple patient cohorts.^23^ This dose‐escalation phase 1 trial enrolled patients with solid tumours harbouring *ROS1*, *NTRK* or *ALK* fusions and established a recommended phase 2 dose (RP2D) of repotrectinib 160 mg once daily for 14 days, followed by 160 mg twice daily. The phase 2 registrational trial focused on patients presenting either *ROS1* or *NTRK* fusions and included 4 distinct cohorts of patients with advanced *ROS1*‐rearranged NSCLC based on prior treatment history: ROS1 TKI‐naïve (EXP‐1), 1 prior ROS1 TKI and chemotherapy‐pretreated (EXP‐2), 2 prior ROS1 TKIs and chemotherapy‐naïve (EXP‐3) and 1 prior ROS1 TKI and chemotherapy‐naïve (EXP‐4). The primary efficacy analysis for *ROS1* fusions was limited to patients who were either ROS1 TKI‐naïve (EXP‐1) or who had received 1 prior ROS1 TKI and were chemotherapy‐naïve (EXP‐4). The primary endpoint of the phase 2 trial was confirmed RECIST version 1.1 objective response as evaluated by blinded independent central review and secondary endpoints included duration of response (DOR), clinical benefit, PFS, OS and intracranial response.

The primary efficacy analysis included 127 patients with *ROS1* fusion‐positive NSCLC: 71 patients were ROS1 TKI‐naïve and 56 patients had received 1 prior ROS1 TKI but were chemotherapy‐naïve. In TKI‐naïve patients, confirmed objective responses were observed in 79% (*n* = 56). With a median follow‐up of 24.0 months, the median DOR was 34.1 months, the median PFS was 35.7 months and the 18‐month OS was 88% (median not reached). In patients who had previously received a ROS1 TKI (crizotinib [*n* = 46], entrectinib [*n* = 9] or ceritinib [*n* = 1]) but were chemotherapy‐naïve, 38% (*n* = 21) had a confirmed objective response. With a median follow‐up of 21.5 months, the median DOR was 14.8 months, the median PFS was 9.0 months and the median OS was 25.1 months. In patients with prior ROS1 TKI treatment who presented a G2032R mutation at baseline (*n* = 17), objective responses were observed in 59% (*n* = 10), confirming the activity of repotrectinib against *ROS1* SF mutations. Repotrectinib also demonstrated encouraging CNS activity with few on‐study CNS progression events. In patients with brain metastases at baseline, intracranial responses were observed in 89% of TKI‐naïve patients (*n* = 8/9) and in 38% of patients who previously received a ROS1 TKI but were chemotherapy‐naïve (*n* = 5/13), with a 12‐month intracranial DOR of 83% and 60%, respectively.

A total of 426 patients treated at the RP2D of repotrectinib were included in the safety analysis. Most adverse events were grade 1 or 2 in severity. Twenty‐nine per cent of patients (*n* = 122) presented grade ≥3 treatment‐related adverse events (AEs) among which anaemia (*n* = 16), increased blood creatine kinase levels (*n* = 15), dizziness (*n* = 11), weight increase (*n* = 7), increased alanine and aspartate aminotransferase (*n* = 6 each) and muscle weakness (*n* = 5) were the most common. Thirty‐eight percent of patients (*n* = 163) required dose reduction, with dizziness reported as the most commonly imputable AE in 11% of cases. AEs led to treatment discontinuation in 7% of patients (*n* = 31) with the most common AE leading to discontinuation being pneumonitis in 1% of cases. Of note, the safety profile of repotrectinib was in line with that of previously approved ROS1/TRK TKIs with neurotoxicity (dizziness, dysgeusia, paresthesia, ataxia, memory impairment) and weight gain likely mediated by concurrent TRK inhibition.^7^, ^9^, ^24^, ^25^ Off‐target inhibition of the JAK/STAT signalling pathway by repotrectinib has also previously been reported and could contribute to the emergence of cytopenias.^19^

In TRIDENT‐1, acquired resistance alterations on repotrectinib included *ROS1* L2086F missense mutation, which occurs on the bottom floor of the ATP binding pocket and involves the catalytic‐spine 6 (CS6) residue of ROS1.^26^ Although less characterized than SF mutations, *ROS1* L2086F has previously been described in the post‐lorlatinib^15^ and post‐taletrectinib setting.^27^ Structural models predicted L2086F mutations would cause steric clashes with crizotinib, entrectinib and lorlatinib,^15^ as well as next‐generation ROS1 TKIs such as taletrectinib and repotrectinib.^26^, ^28^ *ROS1* L2086F mutations however retain sensitivity to type II TKI cabozantinib, with early case reports supporting its efficacy in the clinical setting.^15^, ^26^, ^29^ Additional type I ROS1 TKIs, such as ceritinib and gilteritinib, are also thought to be active in the setting of *ROS1* L2086F and warrant further investigations.^26^, ^28^

In conclusion, repotrectinib demonstrated durable activity in patients with *ROS1*‐rearranged NSCLC, including those with intracranial disease at baseline or presenting *ROS1* G2032R SF mutations following treatment with early‐generation ROS1 TKI. In the treatment‐naïve setting, repotrectinib was associated with the longest duration of disease control reported for patients with *ROS1*‐driven NSCLC, surpassing that observed with previously approved ROS1 TKIs.^8^, ^10^, ^23^, ^30^, ^31^, ^32^ Based on the results of TRIDENT‐1, repotrectinib obtained FDA regulatory approval for the treatment of patients with *ROS1* fusion‐positive NSCLC who are TKI‐naïve or who previously received 1 ROS1 TKI. Future research efforts will focus on establishing the optimal treatment paradigm for ROS1 inhibitors. Such initiatives include TRIDENT‐3, a phase 3 clinical trial comparing repotrectinib to crizotinib in patients with TKI‐naïve *ROS1*‐driven NSCLC (NCT06140836). In addition, next‐generation selective ROS1 TKIs such as NVL‐520 (NCT05118789) are currently being investigated with the hope of decreasing Trk‐mediated toxicities and improving treatment tolerance.^21^

## CONFLICT OF INTEREST STATEMENT

Antoine Desilets has received travel reimbursement from Astra Zeneca, Regeneron and Exelixis. Matteo Repetto has received travel reimbursement from Sanofi. Alexander Drilon has received honoraria and participated in advisory boards for 14ner/Elevation Oncology, Amgen, Abbvie, ArcherDX, AstraZeneca, Beigene, BergenBio, Blueprint Medicines, Chugai Pharmaceutical, EcoR1, EMD Serono, Entos, Exelixis, Helsinn, Hengrui Therapeutics, Ignyta/Genentech/Roche, Janssen, Loxo/Bayer/Lilly, Merus, Monopteros, MonteRosa, Novartis, Nuvalent, Pfizer, Prelude, Repare RX, Takeda/Ariad/Millenium, Treeline Bio, TP Therapeutics, Tyra Biosciences and Verastem; has declared associated research paid to institution by Pfizer, Exelixis, GlaxoSmithKlein, Teva, Taiho and PharmaMar; has received royalties by Wolters Kluwer; has declared food/beverage from Merck, Puma, Merus and Boehringer Ingelheim; and has received CME honoraria by Answers in CME, Applied Pharmaceutical Science, Inc., AXIS, Clinical Care Options, EPG Health, Harborside Nexus, I3 Health, Imedex, Liberum, Medendi, Medscape, Med Learning, MJH Life Sciences, MORE Health, Ology, OncLive, Paradigm, Peerview Institute, PeerVoice, Physicians Education Resources, Remedica Ltd., Research to Practice, RV More, Targeted Oncology, TouchIME and WebMD.

## ETHICS APPROVAL AND CONSENT TO PARTICIPATE

Not applicable.

## References

[1] Neckameyer WS , Shibuya M , Hsu MT , et al. Proto‐oncogene c‐ros codes for a molecule with structural features common to those of growth factor receptors and displays tissue specific and developmentally regulated expression. Mol Cell Biol. 1986;6:1478‐1486. 3023892 10.1128/mcb.6.5.1478-1486.1986 PMC367673

[2] Drilon A , Jenkins C , Iyer S , et al. ROS1‐dependent cancers — biology, diagnostics and therapeutics. Nat Rev Clin Oncol. 2021;18:35‐55. 32760015 10.1038/s41571-020-0408-9 PMC8830365

[3] Davies KD , Le AT , Theodoro MF , et al. Identifying and targeting ROS1 gene fusions in non–small cell lung cancer. Clin Cancer Res. 2012;18:4570‐4579. 22919003 10.1158/1078-0432.CCR-12-0550 PMC3703205

[4] Davare MA , Henderson JJ , Agarwal A , et al. Rare but recurrent ROS1 fusions resulting from chromosome 6q22 microdeletions are targetable oncogenes in glioma. Clin Cancer Res. 2018;24:6471‐6482. 30171048 10.1158/1078-0432.CCR-18-1052 PMC6295214

[5] Lovly CM , Gupta A , Lipson D , et al. Inflammatory myofibroblastic tumors harbor multiple potentially actionable kinase fusions. Cancer Discov. 2014;4:889‐895. 24875859 10.1158/2159-8290.CD-14-0377 PMC4125481

[6] Wiesner T , He J , Yelensky R , et al. Kinase fusions are frequent in Spitz tumours and spitzoid melanomas. Nat Commun. 2014;5:3116. 24445538 10.1038/ncomms4116 PMC4084638

[7] Shaw AT , Ou S‐HI , Bang Y‐J , et al. Crizotinib in ROS1‐rearranged non–small‐cell lung cancer. New Engl J Med. 2014;371:1963‐1971. 25264305 10.1056/NEJMoa1406766 PMC4264527

[8] Shaw AT , Riely GJ , Bang YJ , et al. Crizotinib in ROS1‐rearranged advanced non‐small‐cell lung cancer (NSCLC): updated results, including overall survival, from PROFILE 1001. Ann Oncol. 2019;30:1121‐1126. 30980071 10.1093/annonc/mdz131 PMC6637370

[9] Drilon A , Siena S , Dziadziuszko R , et al. Entrectinib in <em>ROS1</em>fusion‐positive non‐small‐cell lung cancer: integrated analysis of three phase 1–2 trials. Lancet Oncol. 2020;21:261‐270. 31838015 10.1016/S1470-2045(19)30690-4 PMC7811790

[10] Drilon A , Chiu CH , Fan Y , et al. Long‐term efficacy and safety of entrectinib in ROS1 fusion‐positive NSCLC. JTO Clin Res Rep. 2022;3:100332. 35663414 10.1016/j.jtocrr.2022.100332 PMC9160474

[11] Patil T , Smith DE , Bunn PA , et al. The incidence of brain metastases in stage IV ROS1‐rearranged non‐small cell lung cancer and rate of central nervous system progression on crizotinib. J Thorac Oncol. 2018;13:1717‐1726. 29981925 10.1016/j.jtho.2018.07.001 PMC6204290

[12] Park S , Ahn BC , Lim SW , et al. Characteristics and outcome of ROS1‐positive non‐small cell lung cancer patients in routine clinical practice. J Thorac Oncol. 2018;13:1373‐1382. 29883837 10.1016/j.jtho.2018.05.026

[13] Gainor JF , Tseng D , Yoda S , et al. Patterns of metastatic spread and mechanisms of resistance to crizotinib in ROS1‐positive non‐small‐cell lung cancer. JCO Precis Oncol. 2017:2017. 10.1200/PO.17.00063 PMC5766287 29333528

[14] Dziadziuszko R , Hung T , Wang K , et al. Pre‐ and post‐treatment blood‐based genomic landscape of patients with ROS1 or NTRK fusion‐positive solid tumours treated with entrectinib. Mol Oncol. 2022;16:2000‐2014. 35338679 10.1002/1878-0261.13214 PMC9120896

[15] Lin JJ , Choudhury NJ , Yoda S , et al. Spectrum of mechanisms of resistance to crizotinib and lorlatinib in ROS1 fusion‐positive lung cancer. Clin Cancer Res. 2021;27:2899‐2909. 33685866 10.1158/1078-0432.CCR-21-0032 PMC8127383

[16] Awad MM , Katayama R , McTigue M , et al. Acquired resistance to crizotinib from a mutation in CD74–ROS1. New Engl J Med. 2013;368:2395‐2401. 23724914 10.1056/NEJMoa1215530 PMC3878821

[17] Katayama R , Shaw AT , Khan TM , et al. Mechanisms of acquired crizotinib resistance in ALK‐rearranged lung cancers. Sci Transl Med. 2012;4. 120ra17‐120ra17. 10.1126/scitranslmed.3003316 PMC3385512 22277784

[18] Shaw AT , Solomon BJ , Besse B , et al. ALK resistance mutations and efficacy of lorlatinib in advanced anaplastic lymphoma kinase‐positive non–small‐cell lung cancer. J Clin Oncol. 2019;37:1370‐1379. 30892989 10.1200/JCO.18.02236 PMC6544460

[19] Drilon A , Ou SI , Cho BC , et al. Repotrectinib (TPX‐0005) is a next‐generation ROS1/TRK/ALK inhibitor that potently inhibits ROS1/TRK/ALK solvent‐front mutations. Cancer Discov. 2018;8:1227‐1236. 30093503 10.1158/2159-8290.CD-18-0484

[20] Murray BW , Rogers E , Zhai D , et al. Molecular characteristics of repotrectinib that enable potent inhibition of TRK fusion proteins and resistant mutations. Molecular Cancer Therapeutics. 2021;20:2446‐2456. 34625502 10.1158/1535-7163.MCT-21-0632 PMC9762329

[21] Drilon A , Horan JC , Tangpeerachaikul A , et al. NVL‐520 is a selective, TRK‐sparing, and brain‐penetrant inhibitor of ROS1 fusions and secondary resistance mutations. Cancer Discovery. 2023;13:598‐615. 36511802 10.1158/2159-8290.CD-22-0968 PMC9975673

[22] Yun MR , Kim DH , Kim S‐Y , et al. Repotrectinib exhibits potent antitumor activity in treatment‐naïve and solvent‐front–mutant ROS1‐rearranged non–small cell lung cancer. Clinical Cancer Research. 2020;26:3287‐3295. 32269053 10.1158/1078-0432.CCR-19-2777 PMC10283448

[23] Drilon A , Camidge DR , Lin JJ , et al. Repotrectinib in ROS1 fusion–positive non–small‐cell lung cancer. New Engl J Med. 2024;390:118‐131. 38197815 10.1056/NEJMoa2302299 PMC11702311

[24] Mason BL , Lobo MK , Parada LF , et al. Trk B signaling in dopamine 1 receptor neurons regulates food intake and body weight. Obesity. 2013;21:2372‐2376. 23512795 10.1002/oby.20382 PMC3742719

[25] Liu D , Flory J , Lin A , et al. Characterization of on‐target adverse events caused by TRK inhibitor therapy. Ann Oncol. 2020;31:1207‐1215. 32422171 10.1016/j.annonc.2020.05.006 PMC8341080

[26] Thawani R , Repetto M , Keddy C , et al. TKI type switching overcomes ROS1 L2086F in ROS1 fusion‐positive cancers. bioRxiv:2024. 2024.01.16.575901. 10.1038/s41698-024-00663-1 PMC11310217 39117775

[27] Papadopoulos KP , Borazanci E , Shaw AT , et al. U.S. phase I first‐in‐human study of taletrectinib (DS‐6051b/AB‐106), a ROS1/TRK inhibitor, in patients with advanced solid tumors. Clin Cancer Res. 2020;26:4785‐4794. 32591465 10.1158/1078-0432.CCR-20-1630

[28] Ou S‐HI , Hagopian GG , Zhang SS , et al. Comprehensive review of ROS1 tyrosine kinase inhibitors‐classified by structural designs and mutation spectrum (solvent front mutation [G2032R] and central β‐sheet 6 [Cβ6] mutation [L2086F]). J Thorac Oncol. 2024;19(5):706‐718. 38070596 10.1016/j.jtho.2023.12.008

[29] Sakamoto M , Patil T . Exceptional response to lorlatinib and cabozantinib in ROS1‐rearranged NSCLC with acquired F2004V and L2086F resistance. Npj Precis Oncol. 2023;7:56. 37291202 10.1038/s41698-023-00381-0 PMC10250337

[30] Wu YL , Yang JC , Kim DW , et al. Phase II study of crizotinib in East Asian patients with ROS1‐positive advanced non‐small‐cell lung cancer. J Clin Oncol. 2018;36:1405‐1411. 29596029 10.1200/JCO.2017.75.5587

[31] Lim SM , Kim HR , Lee JS , et al. Open‐label, multicenter, phase II study of ceritinib in patients with non‐small‐cell lung cancer harboring ROS1 rearrangement. J Clin Oncol. 2017;35:2613‐2618. 28520527 10.1200/JCO.2016.71.3701

[32] Shaw AT , Solomon BJ , Chiari R , et al. Lorlatinib in advanced ROS1‐positive non‐small‐cell lung cancer: a multicentre, open‐label, single‐arm, phase 1–2 trial. Lancet Oncol. 2019;20:1691‐1701. 31669155 10.1016/S1470-2045(19)30655-2

